# Scaling up electrically synchronized spin torque oscillator networks

**DOI:** 10.1038/s41598-018-31769-9

**Published:** 2018-09-07

**Authors:** Sumito Tsunegi, Tomohiro Taniguchi, Romain Lebrun, Kay Yakushiji, Vincent Cros, Julie Grollier, Akio Fukushima, Shinji Yuasa, Hitoshi Kubota

**Affiliations:** 1 Spintronics Research Center, National Institute of Advanced Industrial Science And Technology (AIST), Tsukuba, 305-8568 Japan; 2 Unité Mixte de Physique CNRS, Thales, Univ. Paris Sud, Université Paris-Saclay, 91767 Palaiseau, France; 3 Institute for Physics, Johannes Gutenberg-University Mainz, 55099 Mainz, Germany

## Abstract

Synchronized nonlinear oscillators networks are at the core of numerous families of applications including phased array wave generators and neuromorphic pattern matching systems. In these devices, stable synchronization between large numbers of nanoscale oscillators is a key issue that remains to be demonstrated. Here, we show experimentally that synchronized spin-torque oscillator networks can be scaled up. By increasing the number of synchronized oscillators up to eight, we obtain that the emitted power and the quality factor increase linearly with the number of oscillators. Even more importantly, we demonstrate that the stability of synchronization in time exceeds 1.6 milliseconds corresponding to 10^5^ periods of oscillation. Our study demonstrates that spin-torque oscillators are suitable for applications based on synchronized networks of oscillators.

## Introduction

Network systems comprising synchronized nonlinear oscillators^1^ are attracting much attention from the perspectives of nonlinear science and practical applications. Among such system, a phased array^2^ for a wave generator or a pattern matching^3^ device consisting of neural networks have been studied so far. The degrees of beam divergence^2^ and pattern matching^3^ are characterized by the phase differences of the synchronized oscillators. Therefore, synchronizing a large number of oscillators and stabilizing their phase differences for a long period of time are critical requirements from the perspective of designing phased-array generators and neural-network-based devices. A spin torque oscillator (STO)^4–6^ is an appropriate candidate for these devices because of its ability to be synchronized due to its strong nonlinear characteristics^7–9^ and advantage in terms of miniaturizing the integrated system due to its small size^10^. Although mutual synchronizations of STOs originating from magnetic coupling^11–18^, which are spin wave propagation^13,14,16,17^ or dipole interaction^10–12,15^, have been reported, it is difficult to simultaneously synchronize a large number of oscillators because the spatial decay of magnetic interactions takes place rapidly within the micrometer range^19^. Recently, we demonstrated synchronization of two STOs by using electrical coupling^20^. In principle, electrical coupling is not restricted by spatial distance; therefore, it has an advantage over magnetic interaction in regard to exciting simultaneous synchronization in an array of a large number of oscillators. Extending the network from two to many oscillators requires a strong coupling to overcome the distribution of natural frequencies in oscillators and disturbances by thermal fluctuation. In this study, we address these crucial issues and realize phase-synchronization of up to eight STOs. The strategy is to fabricate vortex-type STOs having high emission power and high-frequency stability, and to independently control the frequency of each oscillator. Linear dependence of emission power (reaching a maximum value of about 14 µW) on the number of STOs indicates that the phase difference between the STOs is zero, i.e. an in-phase synchronization is excited and not a master-slave synchronization regime. We also found that the in-phase state between two STOs at room temperature remains stable for longer than one millisecond, tha is, 10^5^ periods of oscillation. These results show the great potential of an array of phase-synchronized STOs for a phased-array wave generator and neuromorphic architectures.

### Scalability of emission power

STOs can be electrically synchronized when the interaction between oscillators overcomes their thermal noise and the differences in the natural frequencies^9^. A high emission power from an STO leads to a strong coupling to the other oscillators through the radio-frequency (RF) emitted current. Therefore, as for developing a network of oscillators, high emission power, narrow linewidth and small dispersion of the natural frequency between the STOs are necessary. In this study, magnetic-vortex STOs^21,22^ were used. The STOs typically generate rf signals with power of over 1 μW due to their high magnetoresistance ratio^23,24^. Our previous studies^23,24^ showed that these STOs also have a narrow linewidth (full width at half maximum; FWHM) with a carrier frequency (*f*_STO_) corresponding to a high-quality factor (*f*_STO_/FWHM) of over 2000 due to their large volume. (See Section 1 in Supplementary for details.) In addition, a mutual-synchronization probing system which decreases the dispersion of the natural frequency of the STOs (See Section 2 in Supplementary for details.) was used. In this section, the relation between phase-synchronization and the coupling strength between two oscillators is described first. Next, the scale up of two STOs to eight oscillators is described. It is noteworthy that this number is currently the largest number of synchronized STOs using long-range electrical coupling. It is also noteworthy that the array of eight oscillators generates a huge emission power of 14.1 μW and narrow linewidth of 54 kHz. That emission power is the largest value achieved by using STOs reported to date.

**Figure 1:**
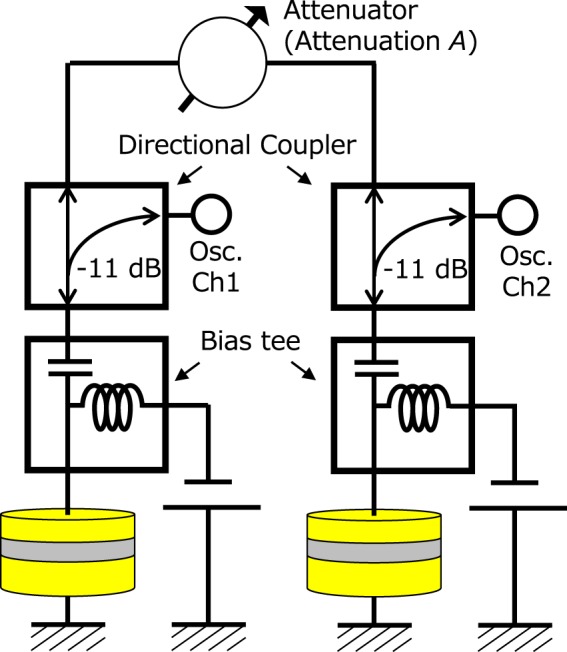
Schematic view of the circuit for measuring the phase difference and merged power between two STOs.

**Figure 2:**
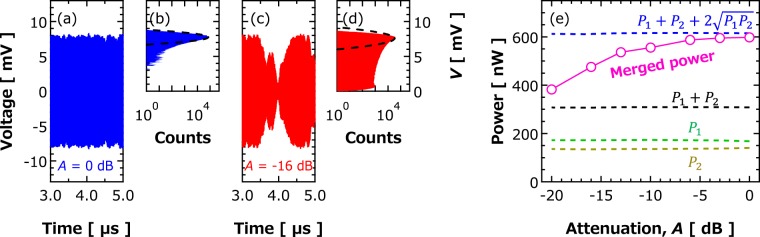
(**a**) Merged voltage of two STOs for attenuation strong coupling, *A* = 0 dB. Individual voltages are measured using the circuit shown in Fig. 1 for 1.6 ms and ideally merged in digital processing. (**b**) Histogram of the envelope of the merged signal shown in (**a**). The dotted line is the result of Gaussian fitting. When the coupling is strong, *A* = 0 dB (blue), a homogeneous signal was obtained. In that case, the histogram shows a Gaussian-like shape and that the voltage amplitude did not decrease down to 0 V. (**c**) Merged voltage and (**d**) its histogram for attenuation of weak coupling, *A* = −16 dB. (**e**) Dependence of emission power of merged signal on attenuation. Here, *P*_1_and *P*_2_ are the emission powers of each STO, whereas ${P}_{1}+{P}_{2}+2\sqrt{{P}_{1}{P}_{2}}$ and *P*_1_ + *P*_2_ are synchronous and asynchronous merged powers, respectively.

An electrically-coupled oscillator network consisting of two STOs with two directional couplers and one attenuator is shown in Fig. 1. The directional couplers are introduced to collect the RF signal generated by individual STOs by using a two-channel oscilloscope. The attenuator (*A*), on the other hand, makes it possible to control the strength of the electric coupling between two STOs, namely, synchronization force and locking range. Total RF voltages were calculated as the sum of measured voltages. As shown in Fig. 2(a), the total RF voltage for a strong coupling of *A* = 0 dB (zero attenuation) shows a homogeneous oscillation. The histogram of the envelope of the total RF voltage is well described by a Gaussian function with a narrow linewidth (see Fig. 2(b)). On the contrary, as shown in Fig. 2(c,d), for a weak coupling (large attenuation) of *A* = −16 dB, the total RF voltage becomes inhomogeneous and the envelope largely deviates from the Gaussian function. The magenta circles in Fig. 2(e) summarize the dependence of emission power of merged signal on the attenuation. The emission power of each oscillator ${P}_{i}$ passing through directional coupler into the oscilloscope, as well as ${P}_{1}+{P}_{2}+2\sqrt{{P}_{1}{P}_{2}}$ and ${P}_{1}+{P}_{2}$ estimated from them, is also shown by the dotted lines. In an oscillator network consisting of two STOs, the emission power of the merged signal is given by ${P}_{1}+{P}_{2}+2\sqrt{{P}_{1}{P}_{2}}\,\langle \cos \,[\Psi (t)]\rangle$. Here, $\Psi$ is the phase difference between the STOs, and the term in the angle brackets (“<>”) indicates a time-averaged value. When in-phase synchronization is excited ($\Psi (t)=0$), merged power is given as ${P}_{1}+{P}_{2}+2\sqrt{{P}_{1}{P}_{2}}$. On the other hand, when the STOs are in asynchronous state, $\langle \cos (\Psi (t))\rangle =0$, and the emission power is reduced to ${P}_{1}+{P}_{2}$. According to the results shown in Fig. 2(e), we conclude that an in-phase synchronization between STOs is excited in a strong coupling (*A* = 0 dB) limit.

**Figure 3:**
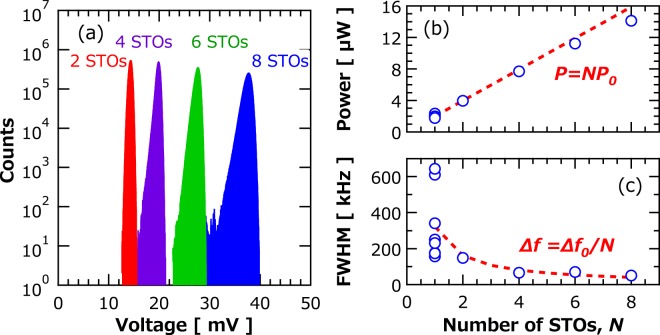
(**a**) Histograms of the envelope of the RF voltage obtained with an array of the several STOs. Dependences of (**b**) emission power and (**c**) spectral linewidth of the STO array on number of oscillators *N*. The results were calculated from the power spectrum of the RF voltages. The dotted red lines in (**b**,**c**) represent *NP0* and Δ*f0*/*N*, here *P0* and Δ*f0* are averaged emission power and linewidth of eight single oscillators measured without coupling, respectively.

To study the stability of phase-synchronization for a large number of STOs, above method was applied. For the increase of the number of STOs, a radial combiner^25^ was introduced in the circuit. Histograms of the envelope of RF voltage generated by STO arrays with 2, 4, 6 or 8 STOs are shown in Fig. 3(a). All the STO arrays clearly show Gaussian-like distributions implying that synchronization between STOs up to eight oscillators is achieved. Dependence of emission power on number of oscillators in the array is shown in Fig. 3(b). It is clear from the figure that emission power increases linearly with increasing number of oscillators, and that result is consistent with the theoretical prediction^26^. Emission power for eight STOs reaches 14.1 μW. The linear dependence of emission power on number of oscillators indicates that in-phase synchronization between eight oscillators is stably realized. The linewidth of the synchronized STOs decreases with increasing number of oscillators;^27^ namely, it is reduced to 54 kHz for eight STOs (See sec. 3 in Supplementary for details), corresponding to high quality-factor (*f*_STO_/FWHM) of 7400 (see Fig. 3(c)). It is emphasized here that the linear dependence of the emission power on the number of the oscillators guarantees the scalability of an oscillator network using STOs.

### Long-term stability of phase-synchronization

As mentioned earlier, one of the critical issues concerning phase-synchronization is to stabilize the phase against thermal fluctuations. Long-term stability is the key to implement oscillator networks in practical applications. For example, pattern recognition requires a stable synchronization longer than 10^3^‒10^4^-times the oscillation period^28^. To give more insights into the phase stability of STOs as a function of time, two coupled vortex-STOs were studied by using the system shown Fig. 1. Phase difference between STOs has been evaluated  by using the Hilbert transform of the RF voltages measured using a high-frequency oscilloscope^29^.

**Figure 4:**
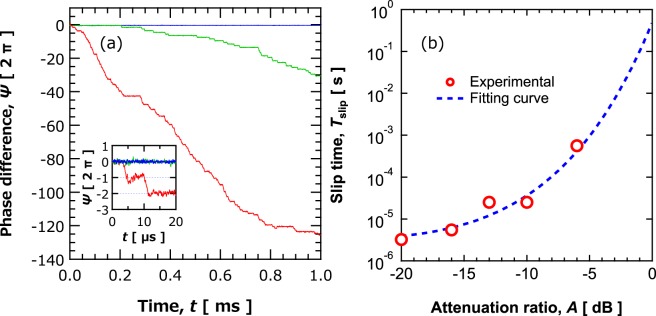
(**a**) Time evolution of the phase difference with attenuations of 0 dB (blue), −10 dB (green), and −16 dB (red). The inset is enlarging view within the time range of 20 *μ*s. Number of phase slips increases with increasing attenuation. Note that the results represent the case that phase difference decreases with time. There is also however a case where phase difference increases with time. The sign of time evolution depends on the detuning of STO natural frequencies. (**b**) Dependence of phase-slip time on attenuation. The red circles are experimentally measured values, whereas the dashed blue line was obtained from theoretically.

Time evolution of the phase difference $\Psi$ between the synchronized STOs for several values of the attenuation *A* is shown in Fig. 4(a). As shown, the phase difference changes with time for the weak coupling (large attenuation) cases. The phenomenon is known as “phase slip”, where the phase difference shifts with the factor of 2 π due to the thermal fluctuation^30,31^ It is noteworthy that a large interval between phase slips corresponds to a long-term stability of phase-synchronization. As shown Fig. 4(a), strong coupling results in an extremely long-term stability of phase difference. The in-phase state is kept for over one millisecond, corresponding to about 10^5^ periods of oscillation for the strongest coupling, i.e., *A* = 0 dB. This result is the first experimental evidence of long-term stability of phase difference in synchronized STOs, and it demonstrates the applicability of STOs to brain-inspired computing schemes such as pattern recognition.

As clearly shown in Fig. 4(a), the phase at *A* = 0 dB is maintained longer than the measurable time limit of the measurement system, which is 1.6 milliseconds. Therefore, the time between phase slips for *A* = 0 dB was theoretically estimated. The phase slip can be regarded as an escape of Brownian particle from a periodic potential with period of 2π. The minima and maxima of the potential locate at 2*n* π and (2*n* + 1) π, respectively (*n* = 0, ±1, ±2, …). Therefore, the phase slip time was calculated as the mean first-passage time^32,33^ (see Section 4 in Supplementary for details). The mean first passage time is a time necessary for a Brownian particle trapped in a potential to escape from it. Evaluating the phase-slip time starts with the equation of motion for the phase, given by^9,31^

11 $$\frac{d{\tilde{\theta }}_{i}}{dt}={\omega }_{i}+\frac{\tilde{F}}{2}\,\sin ({\tilde{\theta }}_{i}-{\tilde{\theta }}_{j}+\beta )+{\xi }_{i}(t),\,i=1,2.$$

Here, ${\tilde{\theta }}_{i}$, ${\omega }_{i}$, $\tilde{F}$, and $\beta$ are the phase of *i*-th STO including the non-linearity^9^, angular velocity, coupling force, and detuning angle^9,34^ corresponding to the sum of the asymmetry of spin transfer torque and the non-linearity, respectively. In our experiments, in-phase synchronization between two STOs was achieved, corresponding to four times the power of the individual STOs after feeding in the connection. This case corresponds to $\beta \, \sim \,0$^20,34^, which gives the largest locking range. White noise ${\xi }_{i}(t)$ satisfies ${\langle \xi }_{i}(t)\rangle =0$ and $\langle {\xi }_{i}(t){\xi }_{j}(t^{\prime} )\rangle =2D\delta (t-t^{\prime} ){\delta }_{i,j}$, where *D* is a diffusion constant corresponding to noise strength being proportional to the linewidth of an STO. From Eq. (1.1), distribution function *S* of phase difference $\Psi ={\tilde{\theta }}_{1}-{\tilde{\theta }}_{2}$ obeys the Fokker-Planck equation^7,9,35^, given as

12 $$\frac{\partial S}{\partial t}=\frac{\partial }{\partial \Psi }[(-\delta \omega +\tilde{F}\,\sin \,\Psi )S]+2D\frac{{\partial }^{2}}{\partial {\Psi }^{2}}S,$$

in which the second term describes diffusion of phase difference *Ψ*. Attenuation *A* is related to the coupling force via $\tilde{F}=\sqrt{A}\tilde{{F}_{0}}$, where $\tilde{{F}_{0}}$ corresponds to the locking range at *A* = 0 dB. The difference between the natural frequencies of the two STOs is $\delta \omega$. For a strong coupling $(\tilde{F}\gg D)$ and small dispersion $(\tilde{F}\gg \delta \omega )$ limits, the time between phase slips is given by

13 $${{\rm T}}_{{\rm{slip}}}=\frac{\pi }{\tilde{F}}{e}^{\frac{\tilde{F}}{D}}.$$

The equation indicates that the time between phase slips is increased exponentially by increasing the coupling force or by decreasing the noise. The time between phase slips evaluated from Fig. 4(a) (red circles) with the one obtained from Eq. (1.3) (blue dashed line) are compared in Fig. 4(b). The fitting parameters, $\tilde{{F}_{0}}$ and $D$, are 3.73 × 10^7^ and 2.4 × 10^6^ rad/s, respectively. These values correspond to locking range of 6 MHz and spectral linewidth of 630 kHz, which are consistent with the experimental results for locking range of a few megahertz and FWHM of 300 kHz. According to the agreement between the experimental results and theoretical results shown in Fig. 4(b), phase-slip time for *A* = 0 dB is predicted to be 0.48 seconds, which corresponds to 10^8^ periods of oscillation. It is noteworthy that this result is the first highlighting a considerable long-term stability of the phase difference between two synchronized (noisy) nano-oscillators.

**Figure 5:**
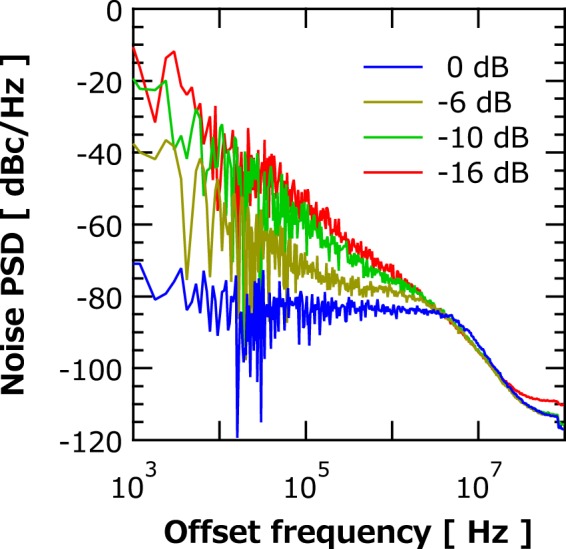
Noise power spectral density (PSD) of the phase difference of synchronized STOs for several values of attenuation.

It should also be noted that the stability of the phase difference described above is comparable to that of forced synchronization^31^. Noise power spectrum density (PSD) of the phase difference for several values of attenuation is shown in Fig. 5. The noise PSD of phase difference is about −20 dBc/Hz at 1 kHz offset for a large attenuation (weak coupling), −16 dB. This value is virtually identical to the phase noise in the case of a single STO^36,37^. The noise PSD decreases to −80 dBc/Hz at 1 kHz offset frequency for the zero attenuation (strong coupling). This value is a great advance toward that of noise PSD attained in the forced synchronization experiment of −90 dBc/Hz^31^. The result suggests that our STO network attains sufficiently stable phase-synchronization without resorting to using an external stabilizer.

### Perspectives: reducing the frequency dispersion of STOs

A mutual synchronization probing system was developed and used for individually controlling STO frequency, and in this manner, a large number of STOs could be electrically synchronized. To control STO frequencies individually, however, the system has a permanent magnet for each STO. This approach was necessary to reduce the frequency dispersion of different STOs and bring them within the locking range. Indeed, the natural frequencies of current STOs are widely distributed (See Supplementary Section 5) due to the fabrication process (which causes wide distribution in size and shape of oscillators).

The critical value of coupling force that is required to realize phase-synchronization in the presence of the frequency dispersion is quantitatively discussed hereafter. An array consisting of *N* oscillators is considered, and the natural frequency of the oscillators is dispersed. Equation (1.1) is extended for *N* oscillators as follows:

21 $$\frac{d\tilde{{\theta }_{i}}}{dt}={\omega }_{i}+\frac{\tilde{F}}{N}\sum _{j\ne i}^{N}\,\sin (\tilde{{\theta }_{i}}-\tilde{{\theta }_{j}})+{\xi }_{i}(t),\,i=1,\ldots ,\,N.$$

Scaling factor *N* is obtained by dividing one oscillator’s coupling force by another’s in the array. Assuming that the distribution of the oscillation frequency is given by a Gaussian function, the critical value of the coupling force for synchronizing the oscillators evaluated from mean-field theory is given by^38^

22 $$\tilde{{F}_{C}}=\frac{\sigma }{\sqrt{\frac{\pi }{8}}\exp (\frac{{D}^{2}}{2{\sigma }^{2}})erfc(\frac{D}{\sqrt{2}\sigma })}$$

where *erfc* denotes the complementary error function and $\sigma$ is the dispersion of the oscillation frequency. The value of *D* is typically in the order of 10^6^ rad/s in the case of our vortex-STOs. The value of $\sigma$ strongly depends on the dispersions of the size/shape of the nano-pillars and the thickness of the free layer. The dispersion of the natural frequency is about 4.6 × 10^7^ rad/s (see Section 5 in Supplementary for details).The critical value of the coupling force necessary to synchronize the oscillators is then estimated to be $\tilde{{F}_{{\rm{C}}}}\,=\,$7  MHz, which is 20 times larger than the experimentally measured value. This fact indicates that the mutual synchronization between STOs cannot be realized in the absence of the probing system. On the other hand, by using the mutual probing system, it is possible to suppress the dispersion to 1.3 × 10^6^ rad/s. Consequently, the critical value of the coupling force decreases down to 3.6 MHz, which is well below that used in our experiments. Therefore, the eight oscillators in the system could be mutually synchronized. The result indicates that the number of STOs can be further increased by controlling their frequency dispersion by refining the quality of their materials and their fabrication process.

## Conclusion

Phase-synchronization of a large number of vortex-type spin torque oscillators (STOs) through interaction via their RF emitted currents was experimentally demonstrated. The number of STOs was increased up to eight. The linear dependence of the emission power from the STO network indicates that the in-phase synchronizations between eight STOs was achieved. The result indicates that the STO network can be scaled up. In an oscillator network consisting of two STOs, long-term stability of the phase difference over 1.6 milliseconds, corresponding to oscillations over 10^5^ periods, was also demonstrated. We note that the measured value of 1.6 milliseconds is the limit of the measurable time in the experimental system, and therefore, it is expected that the phase stability maintains more. Theoretical prediction suggests that the phase stability can be maintained for nearly one second. This long-term stability suppresses the noise power spectral density of phase difference to −80 dBc/Hz at 1 kHz offset frequency. A wide of advantages obtained for coupled oscillators network in this study bring the evidence of the possibility of developing scalable oscillator network for phased array generator and neural network system, such as pattern matching architectures.

## Methods

The complete stack and structure of the STOs used in this work is shown in Supplementary Section 1. The permanent magnet in the probing system applies perpendicular magnetic field to the film plane of up to 6 kOe, which is strong enough to induce gyrotropic motion of the magnetizations. The RF components used are listed as follows: bias-tees (BT), d.c. sources, directional couplers (DC), attenuators, radial combiners (RC) and an oscilloscope (OSC). The BT, DC and attenuators were manufactured by Mini-Circuits (models ZX85-12G+, ZFDC-10-1-S+ and BW-KX-2W44+, respectively). The d.c. sources and OSC were manufactured by Keysight Technologies (models B2902A and DSO9104A, respectively). The RC was manufactured by R&K Company Limited (model RC201M601-0 × 0SS). The RC is designed for 2, 4, 6 or 8 ways. The measured RF voltage was captured by OSC for about 1.6 ms with 5 or 10 Gsam/s. Time domain voltages were transformed to time domain amplitude and phase through Hilbert transform or frequency domain through FFT. The spectrum linewidth were calculated by fitting of the power spectrum of the oscillators array.

## Electronic supplementary material

Supplementary information

## References

[1] Watts DJ, Strogatz SH (1998). Collective dynamics of ‘small-world’ networks. Nature.

[2] Sun J, Timurdogan E, Yaacobi A, Hosseini ES, Watts MR (2013). Large-scale nanophotonic phased array. Nature.

[3] Hopfield JJ (1982). Neural networks and physical systems with emergent collective computational abilities. Proceedings of the National Academy of Sciences.

[4] Kiselev SI (2003). Microwave oscillations of a nanomagnet driven by a spin-polarized current. Nature.

[5] Rippard WH, Pufall MR, Kaka S, Russek SE, Silva TJ (2004). Direct-Current Induced Dynamics in Co_90_Fe_10_/Ni_80_Fe_20_ Point Contacts. Physical Review Letters.

[6] Grollier J, Querlioz D, Stiles MD (2016). Spintronic Nanodevices for Bioinspired Computing. Proceedings of the IEEE.

[7] Pikovsky A., Rosenblum M. & Kurths J. *Synchronization: a universal concept in nonlinear sciences*, vol. 12. (Cambridge university press 2003).

[8] Rippard W (2005). Injection Locking and Phase Control of Spin Transfer Nano-oscillators. Physical Review Letters.

[9] Slavin A, Tiberkevich V (2009). Nonlinear Auto-Oscillator Theory of Microwave Generation by Spin-Polarized Current. Ieee Trans. Magn..

[10] Chung S. W., *et al*. International Electron Devices Meeting, Technical Digest; p. 27. 1 2016. 10.1109/IEDM.2018.8614503 PMC6400221 30846889

[11] Kaka S (2005). Mutual phase-locking of microwave spin torque nano-oscillators. Nature.

[12] Mancoff FB, Rizzo ND, Engel BN, Tehrani S (2005). Phase-locking in double-point-contact spin-transfer devices. Nature.

[13] Ruotolo A (2009). Phase-locking of magnetic vortices mediated by antivortices. Nat Nanotechnol.

[14] Locatelli N (2011). Dynamics of two coupled vortices in a spin valve nanopillar excited by spin transfer torque. Applied Physics Letters.

[15] Sani S (2013). Mutually synchronized bottom-up multi-nanocontact spin–torque oscillators. Nat Commun.

[16] Demidov VE (2014). Synchronization of spin Hall nano-oscillators to external microwave signals. Nat Commun.

[17] Houshang A (2016). Spin-wave-beam driven synchronization of nanocontact spin-torque oscillators. Nat Nano.

[18] Awad AA (2017). Long-range mutual synchronization of spin Hall nano-oscillators. Nat Phys.

[19] Belanovsky AD (2013). Numerical and analytical investigation of the synchronization of dipolarly coupled vortex spin-torque nano-oscillators. Applied Physics Letters.

[20] Lebrun R (2017). Mutual synchronization of spin torque nano-oscillators through a long-range and tunable electrical coupling scheme. Nat Commun.

[21] Pribiag VS (2007). Magnetic vortex oscillator driven by d.c. spin-polarized current. Nature Physics.

[22] Dussaux A (2010). Large microwave generation from current-driven magnetic vortex oscillators in magnetic tunnel junctions. Nat Commun.

[23] Tsunegi S (2014). High emission power and Q factor in spin torque vortex oscillator consisting of FeB free layer. Applied Physics Express.

[24] Tsunegi S, Yakushiji K, Fukushima A, Yuasa S, Kubota H (2016). Microwave emission power exceeding 10 μW in spin torque vortex oscillator. Applied Physics Letters.

[25] Fathy AE, Sung-Woo L, Kalokitis D (2006). A simplified design approach for radial power combiners. IEEE Transactions on Microwave Theory and Techniques.

[26] Georges B, Grollier J, Cros V, Fert A (2008). Impact of the electrical connection of spin transfer nano-oscillators on their synchronization: an analytical study. Applied Physics Letters.

[27] Chang H-C, Cao X, Mishra UK, York RA (1997). Phase noise in coupled oscillators: Theory and experiment. IEEE Trans Microw Theory Tech.

[28] Kumar A, Mohanty P (2017). Autoassociative Memory and Pattern Recognition in Micromechanical OscillatorNetwork. Scientific Reports.

[29] Keller MW, Kos AB, Silva TJ, Rippard WH, Pufall MR (2009). Time domain measurement of phase noise in a spin torque oscillator. Applied Physics Letters.

[30] Finocchio G, Carpentieri M, Giordano A, Azzerboni B (2012). Non-Adlerian phase slip and nonstationary synchronization of spin-torque oscillators to a microwave source. Physiscal Review B.

[31] Lebrun R (2015). Understanding of Phase Noise Squeezing Under Fractional Synchronization of a Nonlinear Spin Transfer Vortex Oscillator. Physical Review Letters.

[32] Risken H. *The Fokker-Planck Equation*, vol. 2nd edition. Springer, 1989.

[33] Gardiner C. *Stochastic Methods*, vol. 4th edition. Springer, 2010.

[34] Tiberkevich V, Slavin A, Bankowski E, Gerhart G (2009). Phase-locking and frustration in an array of nonlinear spin-torque nano-oscillators. Applied Physics Letters.

[35] Kuramoto Y. *Chemical oscillations, waves and turbulence*. Springer-Verlag: Berlin, 1984.

[36] Quinsat M (2010). Amplitude and phase noise of magnetic tunnel junction oscillators. Applied Physics Letters.

[37] Grimaldi E (2014). Response to noise of a vortex based spin transfer nano-oscillator. Physical Review B.

[38] Strogatz SH, Mirollo RE (1991). Stability of incoherence in a population of coupled oscillators. J. Stats. Phys..

