# Supplementary material for: Scaling up electrically synchronized spin torque oscillator networks
Source: Sci Rep. 2018 Sep 7;8:13475. doi: 10.1038/s41598-018-31769-9 (PMC6128876; doi:10.1038/s41598-018-31769-9)
Supplement: Supplementary file 1 — Supplementary information [file 41598_2018_31769_MOESM1_ESM.docx]

**<Supplementary information>**

Scaling up electrically synchronized spin torque oscillator networks

Sumito Tsunegi^1^, Tomohiro Taniguchi^1^, Romain Lebrun^2^, Kay Yakushiji^1^, Vincent Cros^2^, Julie Grollier^2^, Akio Fukushima^1^, Shinji Yuasa^1^, and Hitoshi Kubota^1^

^1^Institute of Advanced Industrial Science and Technology (AIST),Spintronics Research Center, Tsukuba, 305-8560 Japan.

^2^ Unité Mixte de Physique CNRS, Thales, Univ. Paris Sud, Université Paris-Saclay, 91767 Palaiseau, France.

1. **Spin torque oscillator with vortex free layer and its RF properties**

The structure of a spin torque oscillator (STO) is shown in Fig. S1(a). The prepared STO is composed a layered structure of the following stacked films: bottom electrode/buffer-layers/Pt-Mn(15)/Co_70_Fe_30_(2.5)/Ru(0.9)/Co_60_Fe_20_B_20_(2.2)/Co_70_Fe_30_(0.6)/MgO(1)/Fe_80_B_20_(6.0)/MgO(1)/capping layers (thickness in nm). After all the films were deposited, the sample was annealed at 360ºC under vacuum with an in plane magnetic field of 1 T for one hour to crystallize the CoFeB and FeB electrodes as well as to enhance the exchange-bias field of the pinned layer. The films were then formed into a circular-shaped STO with a diameter of 325 nm by electron-beam lithography argon-ion milling. Because of the thick and large-size free layer, the vortex state is stabilized in the free layer. The set up for measuring radio frequency (RF) transport is shown in Fig. S1(b). A standard d.c. source was used to supply a bias voltage (*V*_dc_) to the STO. The positive voltage corresponds to electrons flowing from the FeB layer to the CoFeB. An out-of-plane magnetic field (*H*_OP_) was applied to the STO during the measurement of the RF oscillation. The magnetic field moves the magnetization in the pinned layer in the perpendicular direction. As a result, a large spin torque pointing to the perpendicular direction is excited on the free layer, leading to stable oscillation of the vortex core.^1^ Typical RF properties of the STO are explained as follows.^2^ Dependence of emission power and resistance on magnetic field are shown in Figs. S1(c) and (d), respectively. Because of large magnetoresistance ratio (120% at 10 mV), high emission power (over 1 μW) was obtained. The resistance of the STO was about 50 Ω. By controlling the size and thickness of the MgO barrier, the resistance value was chosen to match the impedance of other RF components, such as probes and cables. Dependences of emission power and linewidth on bias voltage are shown in Figs. S1(e) and(f), respectively. Emission power increases with increasing bias voltage, because the dc current and the amplitude of the trajectory of the gyrotropic motion of the vortex core are increased. Linewidth was typically minimized to 100 kHz at bias voltage of 350‒400 mV.

Figure S1(a) Schematic view of spin torque oscillator. (b) Setup for measuring RF properties of a single STO. Dependences of (c) emission power and (d) resistance of the STO on out-of-plane field *H*_OP_ with bias voltage *V*_dc_ of 400 mV. Dependences of (e) emission power and (f) spectral linewidth (FWHM) on *V*_dc_ for *H*_OP_ =350 mT.


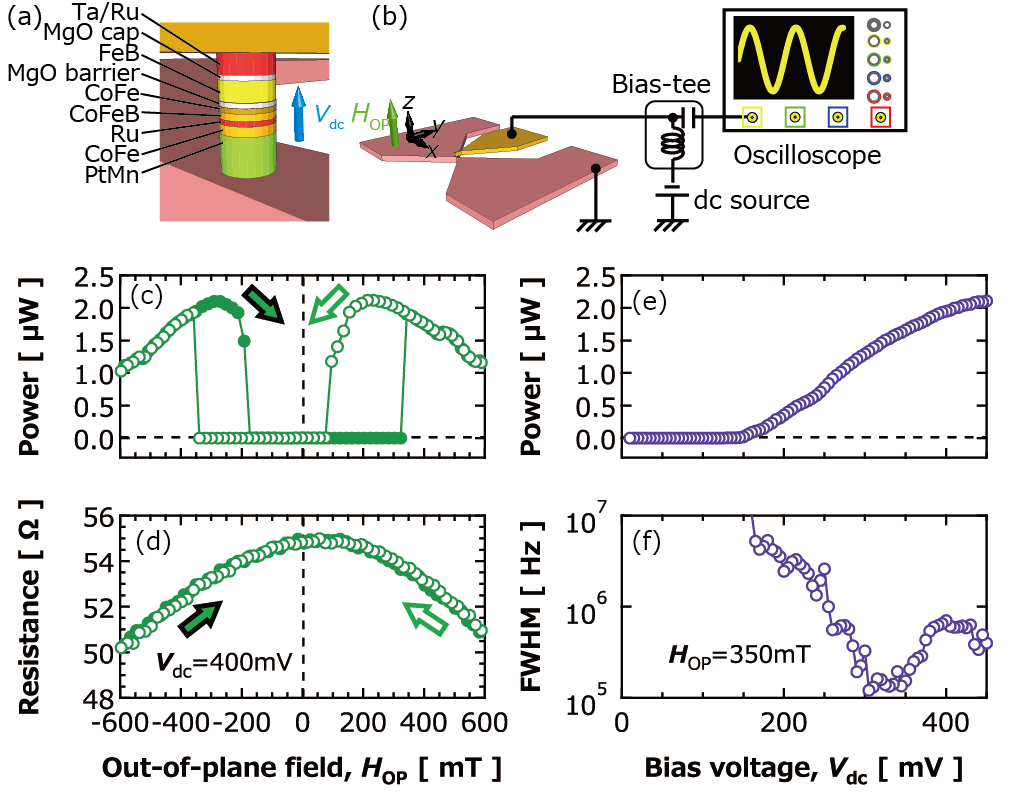


1. **Mutual-synchronization probing system**

It is technically difficult to control STO frequencies individually by using a common dc source and a common magnet. To overcome this problem, the probing system shown Fig. S2 was fabricated and used to control individual STO frequencies. This probing system has permanent magnets and probes for each STO. STO frequency is controlled by tuning the distance between the magnet and the STO and their relative orientations. In addition, it can utilize a variety of RF components and to analyze the RF properties of each STO through each individual probe. As a result, it was possible to analyze the phase difference between the STOs with a directional coupler and the combination of signals with a radial combiner.

Figure S2 The probing system in case of two STOs. It has individual permanent magnets and probes for each STO. STO frequencies are easily tuned by the magnets and the rf signals from the STOs are easily obtained by using the individual probes.


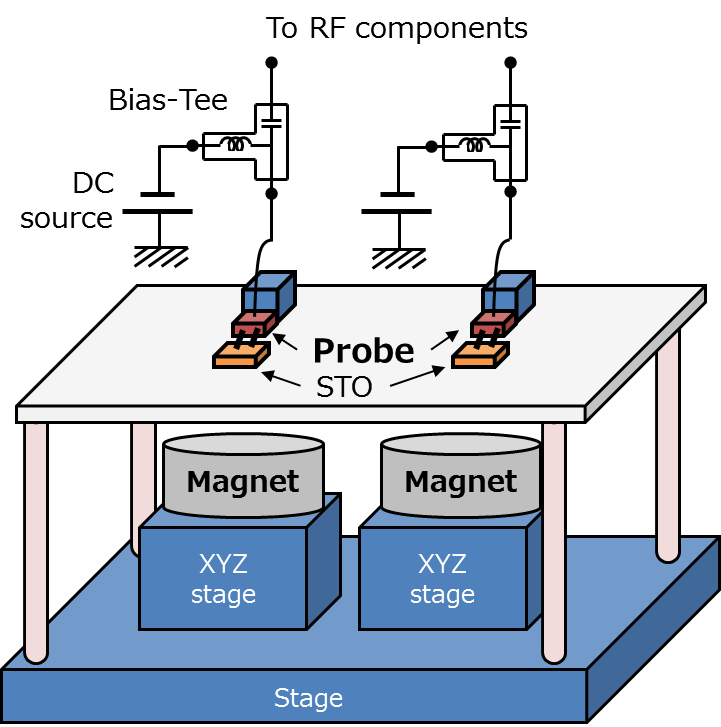


1. **Power spectra of oscillators array**

Typical power spectrum density (PSD) which were transformed from the time domain voltages of individual STOs are shown in Figs. S3(a) and (b). The averaged value of the full width at half maximum (FWHM) among eight STOs is 300 kHz. Typical PSD of the STO arrays are shown in Figs. S3(c) and (d). As a result of the synchronization, FWHM is decreased to 54 kHz for an array of eight STOs.

Figure S3 The PSD of (a), (b)the individual STOs and(c), (d) arrays of STO. Spectra were transformed from time domain to frequency domain of the high-frequency voltages measured in oscilloscope. FWHM were calculated from fitting of the spectra with Lorentzian function.

1. **Theoretical expectation of the phase slip time** $\boldsymbol{T}_{\mathbf{slip}}$

Phase-slip time is derived from the Fokker-Planck equation as follows. As mentioned in the main text, the phase of the *i*-th oscillator obeys the following equation of motion:

|  | $\frac{d\tilde{\theta}_{i}}{dt}=-\omega_{0}+\frac{\tilde{F}}{2}\sin\left( \tilde{\theta}_{i}-\tilde{\theta}_{j} \right)+\xi_{i}\left( t \right),$ | (s1) |
| --- | --- | --- |

where $\omega_{0}$ is assumed to be common between oscillators. The phase difference between oscillators obeys

|  | $\frac{d\Psi}{dt}=\delta\omega-\tilde{F}\sin\Psi+ \xi,$ | (s2) |
| --- | --- | --- |

where $\delta\omega/(2\pi)$ is the difference between the oscillation frequencies of two oscillators. The synchronization occurs when $\left| \frac{\delta\omega}{\tilde{F}} \right|<1$ is satisfied. The dependence of emission power from the STO array on attenuation indicates that the phase difference between the oscillators is almost zero in the synchronized state. This fact means that coupling force $\tilde{F}$ is positive, and a stable fixed point exists at $\Psi_{0}=\sin^{-1} \left( \frac{\delta\omega}{\tilde{F}} \right)$ with $\left| \frac{\delta\omega}{\tilde{F}} \right|\ll1$. On the other hand, unstable fixed points exist at $\Psi_{0}=\pm\pi-\Psi_{0}$. At zero temperature, stochastic force $\xi_{i}$ is zero. In this case, phase difference $\Psi_{0}$ is stably maintained after the phase difference is fixed. At a finite temperature, on the other hand, phase slip from $\Psi_{0}$ to $\pm2\pi+\Psi_{0}$ occurs due to the stochastic force. The phase dynamics at finite temperature is described by the Fokker-Planck equation as

|  | $\frac{\partial S}{\partial t}=\frac{\partial}{\partial\Psi}\left[ \left( -\delta\omega+\tilde{F}\sin\Psi\right)S \right]+2D\frac{\partial^{2}}{\partial\Psi^{2}}S ,$ | (s3) |
| --- | --- | --- |

where *S* is a distribution function, and $D$ is a diffusion constant proportional to the temperature. The steady state solution of Eq. (s3) is

|  | $S\propto\exp\left[ -\frac{V\left( \Psi\right)}{2D} \right] ,$ | (s4) |
| --- | --- | --- |

where $V(\Psi)$ is a potential defined as

|  | $V\left( \Psi\right)=-\delta\omega-\tilde{F}\cos\Psi.$ | (s5) |
| --- | --- | --- |

Comparing Eq. (s4) with the Boltzmann distribution indicates that the phase dynamics can be regarded as motion of a Brownian particle in the potential $V\left( \Psi\right)$, which has minimum at $\Psi_{0}$ and local maxima at $\Psi_{\pm}=\pm\pi-\Psi_{0}$. Phase-slip time is then estimated as a transition time from one minimum$\Psi_{0}$ to the other, $\Psi=\pm2\pi+\Psi_{0}$, by overcoming the potential barrier maximized at $\Psi_{\pm}$. An approach to evaluate the transition time from the Fokker-Planck equation is to calculate mean first-passage time $T_{\mathrm{MFPT}}$^3^ ^4^ ^5^, which is the averaged time for the motion of a Brownian particle from a certain point of a potential to its local maximum. It is evaluated from the backward Fokker-Planck equation, which for the current case is given by

|  | $\frac{d^{2}T_{\mathrm{MFPT}}}{d\Psi^{2}}+\left( \frac{\delta\omega-\tilde{F}\sin\Psi}{2D} \right)\frac{dT_{\mathrm{MFPT}}}{d\Psi}+\frac{1}{2D}=0.$ | (s6) |
| --- | --- | --- |

The absorbing boundary conditions are applied at $\Psi_{\pm}=\pm\pi-\Psi_{0}$. Then, the solution of the mean first-passage time is given by^3^

|  | $T_{\mathrm{MFPT}}\left( \Psi_{0} \right)= \frac{\int_{\Psi_{-}}^{\Psi_{0}} e^{\frac{V\left( \Psi_{1} \right)}{2D}}d\Psi_{1}\int_{\Psi_{0}}^{\Psi_{+}} e^{\frac{V\left( \Psi_{2} \right)}{2D}}d\Psi_{2}\int_{\Psi_{-}}^{\Psi_{2}} e^{-\frac{V\left( \Psi_{3} \right)}{2D}}d\Psi_{3}-\int_{\Psi_{0}}^{\Psi_{+}} e^{\frac{V\left( \Psi_{1} \right)}{2D}}d\Psi_{1}\int_{\Psi_{-}}^{\Psi_{0}} e^{\frac{V\left( \Psi_{2} \right)}{2D}}d\Psi_{2}\int_{\Psi_{-}}^{\Psi_{2}} e^{-\frac{V\left( \Psi_{3} \right)}{2D}}d\Psi_{3}}{2D\int_{\Psi_{-}}^{\Psi_{+}} e^{\frac{V\left( \Psi_{1} \right)}{2D}}d\Psi_{1}} .$ | (s7) |
| --- | --- | --- |

Under the strong coupling limit, the first term of the numerator is dominated.

|  | $T_{\mathrm{MFPT}}\left( \Psi_{0} \right)=\frac{\pi}{\tilde{F}\cos\Psi_{0}}\frac{e^{\frac{V\left( \Psi_{+} \right)}{2D}}e^{\frac{V\left( \Psi_{-} \right)}{2D}}e^{-\frac{V\left( \Psi_{0} \right)}{2D}}}{e^{\frac{V\left( \Psi_{+} \right)}{2D}}+e^{\frac{V\left( \Psi_{-} \right)}{2D}}} .$ | (s8) |
| --- | --- | --- |

When the difference in frequencies of the oscillators is small, $|\delta\omega/K|\ll1$, it can be approximated that ${V(\Psi}_{+})\cong{V(\Psi}_{+})$. Then, the mean first passage time becomes

|  | $T_{\mathrm{MFPT}}\left( \Psi_{0} \right)\cong\frac{\pi}{2\tilde{F}}exp\left( \frac{\tilde{F}}{D} \right).$ | (s9) |
| --- | --- | --- |

On the other hand, when the difference in frequencies of the oscillators is large, it follows that

|  | $T_{\mathrm{MFPT}}\left( \Psi_{0} \right)\cong\frac{\pi}{\tilde{F}\cos\Psi_{0}}\exp\left[ \frac{V\left( \Psi_{+} \right)-V\left( \Psi_{0} \right)}{2D} \right],$ | (s10) |
| --- | --- | --- |

where it is assumed that $\delta\omega>0$, for convention. Note that

|  | $V\left( \Psi_{\mp} \right)-V\left( \Psi_{0} \right)=2\left[ \delta\omega\left( \mp\frac{\pi}{2}+\Psi_{0} \right)+\tilde{F}\cos\Psi_{0} \right]$. | (s11) |
| --- | --- | --- |

Once the Brownian particle arrives at the absorbing point, it moves to neighboring stable states or back to the original one with equal probability, 1/2^6^. Therefore, the phase slip time is given as

|  | $T_{\mathrm{slip}}=2T_{\mathrm{MFPT}}.$ | (s12) |
| --- | --- | --- |

In the main text, it was considered that the difference between frequencies of two oscillators is small; therefore, phase slip time is given by

|  | $T_{\mathrm{slip}}\cong\frac{\pi}{\tilde{F}}\exp\left( \frac{\tilde{F}}{D} \right).$ | (s13) |
| --- | --- | --- |

1. **Distribution of STO frequencies and critical value of coupling force**

The natural frequencies of the STOs are widely distributed during their fabrication process because STO frequency depends on the volume of the free layer. A histogram of the STO frequencies of 205 elements is shown in Fig. S4(a). All frequencies were measured under the same condition: *H*_OP_=600 mT and *V*_dc_ =400 mV. Standard deviation of frequency was 7.3 MHz, and standard deviation (σ) of angular velocity was 46 MHz. A Q–Q (quantile-quantile) plot of STO frequencies are shown in Fig. S4(b). The plot shows linear trends, indicating that the distribution is Gaussian. The critical value of coupling force $\tilde{F_{C}}$ (calculated by using Eq. (2.2) in the main text) is shown in Fig. S5. It is clear that the critical value increases with increasing thermal noise *D*, which is proportional to the spectral linewidth, and the frequency variation σ of frequency of the STOs.

Figure S4 (a) Histogram of STO frequencies measured at *H*_OP_=600 mT and *V*_dc_ =400 mV. Number of elements is 205. The dotted line indicates the Gaussian. The average value is 449 MHz, and the value of standard deviation is 7.3 MHz. (b) Q-Q plot made from the data in S4(a).


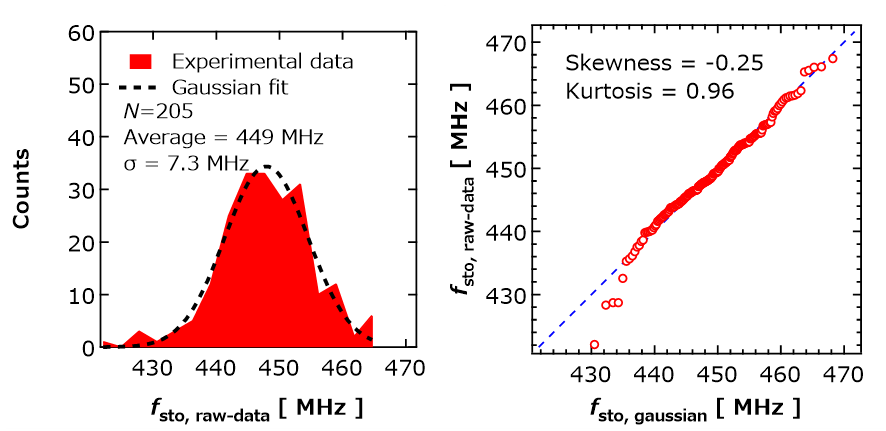


Figure S5 Critical value of coupling force $\tilde{F_{C}}$ calculated by using Eq. (2.2) in the main text. Note that all units are angular velocity.


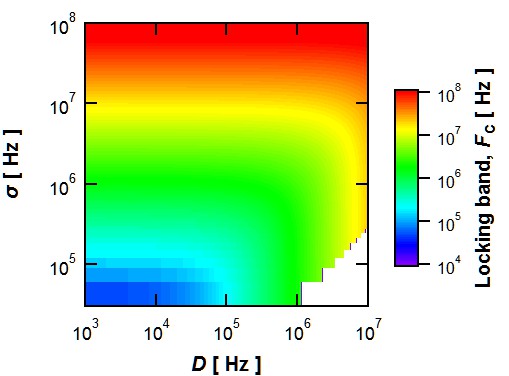


**References**

1 Dussaux, A. *et al.* Large microwave generation from current-driven magnetic vortex oscillators in magnetic tunnel junctions. *Nat Commun* **1**, 8 (2010).

2 Tsunegi, S. *et al.* High emission power and Q factor in spin torque vortex oscillator consisting of FeB free layer. *Applied Physics Express* **7**, 063009 (2014).

3 Risken, H. *The Fokker-Planck Equation*. Vol. 2nd edition (Springer, 1989).

4 Gardiner, C. *Stochastic Methods*. Vol. 4th edition (Springer, 2010).

5 Taniguchi, T. & Imamura, H. Thermal switching rate of a ferromagnetic material with uniaxial anisotropy. *Phys Rev B* **85**, 184403 (2012).

6 Hanggi, P., Talkner P., & Borkovec M., Reaction-rate theory: fifty years after Kramers. *Rev Mod Phys* **62**, 251 (1990).
